# Coelimycin Synthesis Activatory Proteins Are Key Regulators of Specialized Metabolism and Precursor Flux in *Streptomyces coelicolor* A3(2)

**DOI:** 10.3389/fmicb.2021.616050

**Published:** 2021-04-09

**Authors:** Bartosz Bednarz, Aaron Millan-Oropeza, Magdalena Kotowska, Michał Świat, Juan J. Quispe Haro, Céline Henry, Krzysztof Pawlik

**Affiliations:** ^1^Hirszfeld Institute of Immunology and Experimental Therapy, Polish Academy of Sciences, Wrocław, Poland; ^2^PAPPSO, Micalis Institute, INRAE, AgroParisTech, Université Paris-Saclay, Jouy-en-Josas, France

**Keywords:** SARP, antibiotic biosynthesis, specialized metabolites, secondary metabolism, precursor flux, dormancy, stress response, proteomics

## Abstract

Many microbial specialized metabolites are industrially relevant agents but also serve as signaling molecules in intra-species and even inter-kingdom interactions. In the antibiotic-producing *Streptomyces*, members of the SARP (*Streptomyces* antibiotic regulatory proteins) family of regulators are often encoded within biosynthetic gene clusters and serve as their direct activators. Coelimycin is the earliest, colored specialized metabolite synthesized in the life cycle of the model organism *Streptomyces coelicolor* A3(2). Deletion of its two SARP activators *cpkO* and *cpkN* abolished coelimycin synthesis and resulted in dramatic changes in the production of the later, stationary-phase antibiotics. The underlying mechanisms of these phenotypes were deregulation of precursor flux and quorum sensing, as shown by label-free, bottom-up shotgun proteomics. Detailed profiling of promoter activities demonstrated that CpkO is the upper-level cluster activator that induces CpkN, while CpkN activates type II thioesterase ScoT, necessary for coelimycin synthesis. What is more, we show that *cpkN* is regulated by quorum sensing gamma-butyrolactone receptor ScbR.

## Highlights

- Coelimycin activators control stress response, dormancy and other antibiotics.
- CpkO inhibits precursor flux to other pathways.
- CpkN activates type II thioesterase ScoT, necessary for coelimycin synthesis.

## Introduction

*Streptomyces* are soil-dwelling, filamentous bacteria that produce two-thirds of clinically relevant antibiotics (Hopwood, 2007). They undergo a complex life-cycle of vegetative growth, aerial mycelium formation and sporulation, resembling the processes of filamentous fungi (Hopwood, 2007). The genome of the model actinomycete *Streptomyces coelicolor* A3(2) encodes more than 20 specialized metabolite gene clusters (Bentley et al., 2002). Their transcription is induced by environmental, physiological or nutrient-limitation signals coupled with vegetative mycelium autolysis and subsequent salvage of its constituents for aerial mycelium formation and sporulation (Bibb, 2005). Functionally, specialized metabolites can range from means of competition, survival and communication, with many falling into several categories. Not only does the unsurpassed potential of microorganisms to produce multitude of specialized metabolites appear as means for the pharmaceutical industry to overcome the growing antibiotic resistance, but it also poses a question of how these intra- and inter-species communication molecules shape the environment. Moreover, these interactions are the key to unlocking even bigger chemical space for specialized metabolites in a wider array of organisms, as seen from the growing interest in inter-kingdom (bacteria – fungi) co-cultivation experiments (Netzker et al., 2015).

*Streptomyces coelicolor* A3(2) chromosome encodes proteins for the production of four antibiotics: two polyketides – coelimycin A (CPK A) and the blue-colored actinorhodin (ACT), the pyrrole-based red pigment undecylprodigiosin (RED) and the lipopeptide calcium-dependent antibiotic (CDA) (Liu et al., 2013). Each biosynthetic gene cluster (BGC) encodes its own pathway-specific *Streptomyces* antibiotic regulatory proteins (SARPs): CpkO and CpkN (*cpk* cluster), CdaR (*cda* cluster), RedZ and RedD (*red* cluster), and ActII-ORF4 (*act* cluster). Members of SARP family characterized so far are activators and cellular levels of their transcripts generally correlate with respective antibiotic production levels (Takano et al., 1992; Gramajo et al., 1993). These pathway-specific transcription factors are subject to control by pleiotropic regulators associated with multiple mechanisms of primary and specialized metabolism (van der Heul et al., 2018). Although functionally cluster-specific regulators (CSRs) are accepted as “lower-level” regulators and are associated with their respective BGCs, evidence of their influence on other BGCs is increasing (McLean et al., 2019). Earlier transcriptomic studies identified distant genes of *S. coelicolor* A3(2) with expression patterns coordinated with those of biosynthetic gene clusters, namely *ecr* (expression coordinated with *red*) and *eca* (expression coordinated with *act*). What is more, experiments on pathway-specific regulator cross-control revealed that overexpression of *cdaR*, *actII-ORF4* or *redD* decreased the abundance of *cpk* cluster gene transcripts. An indication of a pleiotropic regulator *afsS* activation by *redZ* overexpression was presented in the same work (Huang et al., 2005).

The 58 kb coelimycin biosynthetic gene cluster includes 24 genes (*SCO6265-SCO6288*) which belong to functional groups of: i) core biosynthetic, ii) precursor supply, iii) post-polyketide tailoring, iv) export and v) regulatory genes (Pawlik et al., 2007; Gomez-Escribano et al., 2012). For almost a decade the product of *cpk* cluster remained to be “cryptic.” In 2010, two groups reported detection of a yellow pigment production which was induced in a minimal medium supplemented with glutamate (Gottelt et al., 2010) or in a rich medium lacking glucose (Pawlik et al., 2010). It is first synthesized as a colorless antibiotic associated with mycelium (abCPK; Gottelt et al., 2010), deduced to be a *bis*-epoxide coelimycin A (Challis, 2014), and in the medium it forms adducts with *N*-acetylcysteine or glutamate producing colored coelimycins P1 or P2, respectively, which no longer have antibacterial activity (Gottelt et al., 2010; Gomez-Escribano et al., 2012). The polyketide chain of coelimycin is assembled by the modular polyketide synthase subunits – CpkA, CpkB, and CpkC (Gomez-Escribano et al., 2012). During CPK synthesis, type II thioesterase ScoT removes non-reactive acyl residues blocking the synthase “assembly line.” *scoT* gene was shown to be mandatory for coelimycin synthesis (Kotowska et al., 2014). The current state of knowledge on coelimycin synthesis regulation was recently reviewed by our group (Bednarz et al., 2019). The expression of *cpk* genes is controlled by two SARPs – CpkO (SCO6280) and CpkN (SCO6288) and the butanolide system proteins including: ScbA (SCO6266) – a γ-butyrolactone (GBL) synthase, ScbB (SCO6267) – a butenolide phosphate reductase, ScbR (SCO6265) – a GBL receptor, and ScbR2 (SCO6286) – a pseudo-GBL receptor protein. In its native state, ScbR represses the promoter of the main *cpk* cluster activator gene *cpkO*, but it dissociates from it upon GBL binding, leading to induction of coelimycin synthesis (Takano et al., 2001, 2005). Interestingly, ScbR2 protein does not bind GBLs but antibiotics actinorhodin and undecylprodigiosin instead (Xu et al., 2010). Its function was proposed to be a “late repressor,” switching off *cpk* cluster transcription (Gottelt et al., 2010; Liu et al., 2013). Studies on *S. lividans* TK24 indicated that ScbR2 may bind coelimycin and dissociate from *cpk* promoters, further inducing CPK synthesis (Sun et al., 2017). Our results presented in this work do not support this hypothesis of a positive feedback loop between CPK and its biosynthetic genes transcription.

In order to gain insights into the interaction network between *cpk* regulatory proteins and other specialized metabolite BGCs, we analyzed the proteomes of *S. coelicolor* A3(2) parent strain M145 and deletion mutants Δ*cpkO* (P193 strain) and Δ*cpkN* (P196 strain). The method (bottom-up, label-free shotgun proteomics) along with the instrumentation used (Orbitrap Fusion Lumos Tribrid mass spectrometer, Thermo Scientific) allows for sufficient protein identification levels to portray the changes in the cell throughout different conditions and strains. Furthermore, we performed phenotypic analysis and employed *in vivo* reporter system measurements over 110 h of growth in order to confirm proteomic effects and unravel detailed profiles of *cpk* gene transcription and regulation mechanism. In this work, we also provide evidence for an interplay between butanolide system proteins ScbR and ScbR2 in *cpk* cluster regulation.

## Materials and Methods

### Bacterial Strains, Growth Conditions and DNA Manipulation

Bacterial strains used in this study are listed in Supplementary Table S1. For genetic manipulation, *Escherichia coli* and *Streptomyces coelicolor* A3(2) strains were grown as described previously (Kieser et al., 2000; Sambrook and Russell, 2001). For phenotype, reporter and proteomic studies medium 79 without glucose (79NG) (Pawlik et al., 2010) was used (10 g peptone, 2 g acid hydrolysate of casein, 2 g yeast extract, 6 g NaCl, and 20 g agar for 1,000 ml of medium, pH 7.3). PCR-amplified DNA fragments were first cloned into pGEM-T Easy (Promega) or pTZ57R/T (Thermo Scientific) vector to be verified by DNA sequencing and then cloned into appropriate plasmids. Constructs and oligonucleotides used in this study are listed in Supplementary Tables S2 and S3, respectively.

### *S. coelicolor* A3(2) Deletion, Overexpression and Complementation Mutants

St1G7 cosmid^1^ derivative, in which *cpkO* gene sequence was replaced with an apramycin resistance gene *aac(3)IV* (St1G7-cpkO_DM_), was constructed by means of PCR-targeting (Gust et al., 2003). Cosmid 11B05.G04, in which *cpkN* gene sequence was disrupted with Tn5062 transposon (also containing *aac(3)IV* casette), was obtained thanks to P. J. Dyson (Fernández-Martínez et al., 2011). Complementation construct pIJ10257-cpkN_CO_ was generated by cloning *cpkN* promoter-gene sequence into KpnI-HindIII sites of pIJ10257 (Hong et al., 2005). Complementation construct pIJ10257-cpkO_CO_ was generated by cloning *cpkO* promoter-gene sequence into PvuII site of pIJ10257XermEp plasmid. The constructs (Supplementary Table S2) were introduced into the genomes of the respective *S. coelicolor* A3(2) strains by *E. coli* ET12567/pUZ8002-mediated conjugation.

### Phenotype and Reporter System Studies

For visual imaging of strain phenotypes, 20 μl of respective *S. coelicolor* A3(2) spore suspensions in water (OD_600_ = 0.3) were spotted on solid medium 79NG and grown in 30°C for 114 h. Bacteria were photographed at 18, 21, 25, 41, 48, 70, 96, and 114 h timepoints.

For the promoter activity reporter system assay, a set of pFLUXH (Szafran et al., 2016) derivative plasmids, containing different *cpk* cluster promoter sequences, was prepared (Supplementary Table S2). Respective DNA fragments were PCR-amplified (Supplementary Table S3), cloned into pTZ57R/T plasmid, cut out with appropriate restrictases and cloned into pFLUXH NdeI or BamHI-NdeI sites. pFLUXH derivatives were introduced into respective *S. coelicolor* A3(2) strains by means of conjugation (Supplementary Table S1). A total of 200 μl of solid medium 79NG was poured into optical-bottom, white 96-well plate (Thermo Scientific). A total of 10 μl of spore suspensions (OD_600_ = 0.3) of generated *S. coelicolor* A3(2) strains were inoculated in the wells and grown in ClarioStar Plus microplate reader (BMG Labtech) for 110 h in 30°C. Luminescence was measured automatically every 30 min, the focal height was set to 15 mm and gain was set to 3,600. Each strain containing a reporter construct was plated as three biological and three technical replicates.

### Assay for Calcium Dependent Antibiotic (CDA) Production

A total of 20 μl of *S. coelicolor* A3(2) strains spore suspensions (OD_600_ = 0.3) were spotted on solid medium 79NG (20 ml of medium) and grown at 30°C for 27 h. The plates were then overlayed with 12 ml of soft nutrient agar (1% of agar) (Kieser et al., 2000) containing 32 mM Ca(NO_3_)_2_ mixed with 120 μl of overnight liquid culture of *Bacillus mycoides* PCM2009 indicator strain and incubated for 18 h. In the control plate Ca(NO_3_)_2_ was omitted.

### Purification of ScbR and ScbR2 Proteins

Gene *scbR2* was cloned into NdeI and XhoI sites of pET28a(+) giving plasmid pET28-scbR2. Gene *scbR* was cloned into NdeI and EcoRI sites of pET28a(+) giving plasmid pD01-HN-scbR (Supplementary Tables S2, S3). The expression vectors were used to transform *E. coli* BL21(DE3)pLysS strain. Bacterial cultures in 200 ml of LB medium with kanamycin (30 μg/ml) were shaken (180 rpm) at either 30°C (for ScbR) or 37°C (for ScbR2) until OD_600_ = 0.6. Cultures were induced with 0.1 mM IPTG and incubation was continued for 3 h. Biomass was centrifuged, washed with 20 ml of lysis buffer (50 mM NaH_2_PO_4_, 300 mM NaCl) and frozen at −20°C. Bacterial cells were resuspended in 15 ml of lysis buffer, disrupted by sonication and centrifuged (20,000 × *g*, 30 min, 4°C). A total of 250 μl of His-Select Nickel Affinity Gel (Sigma) was added to clarified lysate and incubated on a roller for 40 min at room temperature. The resin was centrifuged (5,000 × *g*, 5 min), washed five times with lysis buffer, twice with lysis buffer containing 10 mM imidazole and transferred to an empty column. His tagged ScbR and ScbR2 proteins were eluted with lysis buffer containing 250 and 100 mM imidazole, respectively. Glycerol was added to eluate fractions to the final concentration of 50%. Obtained proteins were stored at −20°C.

### Electrophoretic Mobility Shift Assay (EMSA)

Promoter region fragments of *cpkN* gene – pcpkN and pcpkN-a were PCR-amplified using primer pairs NF, NR, and UP1C, NR, respectively. The fragments were then cloned into pTZ57R/T and generated constructs were used as a template for fragment amplification using primers pTZBAM800 and pTZXBA800 complementary to pTZ57R/T sequences flanking the insert. pTZ-MYCO plasmid was used as a template for amplification of an unspecific-binding control DNA fragment MYCO using primers pTZBAM700 and pTZXBA700. Each EMSA sample (volume 16 μl) contained 0.01 pmol of IRDye 800-labeled pcpkN fragment or 0.03 pmol of IRDye 800-labeled fragment pcpkN-a and the same amount of IRDye 700-labeled control fragment MYCO, variable amount of ScbR or ScbR2 protein, 100 ng of herring sperm DNA, 1 μl of 25 mM DTT-2.5% Tween 20 mix and 1.6 μl of 10× LI-COR buffer (100 mM Tris-HCl pH 7.5, 500 mM KCl, and 10 mM DTT). Samples were incubated for 20 min. in the dark at room temperature and then supplemented with 4 μl of 40% sucrose as a loading agent. Samples were resolved on 4% native polyacrylamide gels (composition of a 10 ml gel: 1,33 ml 30% acrylamide-0.8% bisacrylamide solution, 1 ml of 10× TBE buffer, 7.61 ml H_2_O, 7.5 μl TEMED, 75 μl 10% APS). Gels were visualized using Odyssey Imager (LI-COR).

### DNase I Footprinting

Fragments pcpkNup and pcpkN-a were amplified with primer pairs UP1, UP2 and UP1C, NR, respectively. In each pair, one of the primers was labeled on its 5′ end with [^32^P]-ATP at a time using T4 polynucleotide kinase. A total of 20 μl of the binding mixture containing radiolabeled DNA fragment (∼200), variable amount of ScbR/ScbR2 protein and 2 μl of 10× LI-COR buffer was incubated for 10 min in 30°C. Then 5 μl of NaCl and CaCl_2_ mixture was added to the sample to the final concentration of 2 and 1 mM, respectively, followed by addition of 5 μl of DNase I (Thermo Scientific) dilutions and H_2_O to the final volume of 35 μl. Samples were then incubated in 30°C for 5 min. The reaction was stopped by addition of 35 μl of Stop buffer (200 mM NaCl, 100 mM EDTA, 1% SDS) and incubation for 10 min at 75°C. DNA was extracted from the samples using 25:24:1 phenol:chloroform:isoamyl alcohol followed by ethanol precipitation. DNA was then dissolved in Loading buffer (95% formamide, 20 mM EDTA, 0.05% bromophenol blue, 0.05% xylene cyanol FF), resolved on a denaturing 8% polyacrylamide-Tris-borate-EDTA gel and visualized using Typhoon FLA 9500 Imager (GE Healthcare). Dideoxy sequencing ladders were generated using Thermo Sequenase Cycle Sequencing Kit (Affymetrix) and the labeled primers.

### Preparation of Samples for Proteomic Analysis

A total of 200 μl of spore suspensions (OD600 = 0.3) of strains M145, Δ*cpkO* and Δ*cpkN* were streaked on solid medium 79NG overlaid with a perforated cellophane disk. Four biological replicates were prepared for each strain. After 27 h of growth in 30°C, all biomass was scraped off from the surface with a scalpel, washed two times with 50 mM Tris-HCl pH 7.8 and frozen in −80°C. Proteins were extracted and digested as described before (Millan-Oropeza et al., 2017) with small modifications. The pellets were thawed on ice, suspended in 3 ml of DUTT buffer (6 M urea, 2 M thiourea, 5 mM DTT, 0.1 M Tris-HCl pH 8) and 150 μl of protease inhibitor cocktail (Sigma) was added to each sample. Samples were then processed in One Shot Cell Disruptor (Constant Systems LTD) by two disruption shots at 2.6 kBars, cell debris was removed by centrifugation (4,500 × *g*, 15 min, 4°C) and soluble proteins were recovered. Protein concentration in the samples was measured using 2D Quant Kit (GE Healthcare). Aliquots of 50 μg of each protein extract were supplemented with RapiGest^TM^ (Waters) and iodoacetamide to a final concentration of 0.1% and 50 mM, respectively. The samples were then incubated in the dark at room temperature for 45 min (alkylation). Subsequently, 1 μg of lysyl-endopeptidase LysC (Wako) was added to each sample, followed by incubation at 37°C for 3 h. Next, samples were diluted 6× with deionized H_2_O and 1 μg of modified porcine trypsin (Promega) was added, followed by overnight incubation at 37°C. Trifluoroacetic acid was added to adjust pH to 2 to quench the digestion reaction. Peptides were pre-cleaned using Strata-X columns (Phenomenex) by washing with 1.5 ml of washing buffer (3% acetonitrile (ACN), 0.06% glacial acetic acid). Peptides were recovered using 600 μL of elution buffer (40% ACN and 0.06% glacial acetic acid). Samples were then dried under vacuum and resuspended in 320 μl of loading buffer (0.1% trifluoroacetic acid, 2% ACN).

### LC-MS/MS Analysis

A total of 4 μl of each sample (1 μg of peptides) were injected into a Dionex Ultimate 3000 RSLC system coupled to an Orbitrap Fusion^TM^ Lumos^TM^ Tribrid^TM^ mass spectrometer (Thermo). Peptides were separated in an Acclaim PepMap 75 μm (diameter) × 500 mm (length) column packed with 3 μm diameter superficially porous particles (Thermo Scientific). The separation was performed at the flow of 0.3 μl/min with a linear gradient 1–35% (0.1% formic acid and 80% ACN) for 160 min and 35–50% for 10 min. A complete run including regeneration (98% buffer) was of 215 min. Nanospray ionization was performed by applying 1.6 kV in a positive mode. Capillary transfer was performed at 275°C using a capillary probe SilicaTip Emitter 10 μm.

The mass spectrometer was operated in data dependent acquisition mode. Full MS scan occurred in the Orbitrap (scan range 400–1,600 m/z) with a resolution of 120,000 (AGC target of 5 × 10^5^, maximum injection time of 100 ms and data type of centroid). Analyzed charge states were set to 2–5 with a top speed cycle of 3 s for the most intense double or multiple charged precursor ions. The dynamic exclusion was set to 10 ppm, duration of 60 s, and the intensity threshold was fixed at 5 × 10^4^. MS2 was performed using High Collision Dissociation (HCD) in the Orbitrap with the resolution of 15,000 (collision energy of 30 %, AGC target of 5.0 × 10^4^, max. injection time of 150 ms). Polysilaxolane ions m/z 445.12002, 519.13882, 593.15761, and 667.1764 were used for internal calibration.

### Protein Identification and Quantification

Mass spectrometry was analyzed as described before (Millan-Oropeza et al., 2017). Protein identification was performed with X!TandemPipeline C++ 0.2.24 (Langella et al., 2017) using X!Tandem algorithm^2^ (version Alanine 2017.02.01) and the *Streptomyces coelicolor* A3(2) database obtained from UniProt^3^ (17.10.2018). Protein cleavage sites were defined for trypsin, with a maximum of 1 missed cleavage site. Carboxyamidomethylation of cysteine residues and oxidation of methionine residues were set to “fixed” and “potential” modifications, respectively. Precursor mass tolerance and fragment mass tolerance were set to 10 ppm. Data filtering was achieved according to a peptide *E*-value <0.01, protein log (*E*-value) < −4 and to a minimum of two identified peptides per protein. Peptide and protein False Discovery Rates (FDR) were estimated at 0.03 and 0.17%, respectively. MS1 peaks were detected and aligned using MassChroQ 2.2.12 (Valot et al., 2011).

Relative quantification of protein abundances was performed using three complementary methods: spectral counting (SC) defined as the number of MS2 spectra assigned to a protein (Liu et al., 2004), extracted ion chromatograms (XIC) defined as the sum of MS1 intensities of all peptides associated with a protein and peak counting (PC) defined as the number of MS1 chromatogram peaks (peptides) attributed to each protein. XIC method is suitable for detecting subtle differences in protein abundance based on specific peptide data while SC allows only to detect larger abundance variations including that of presence/absence, taking advantage of both specific and shared peptides in our study (Blein-Nicolas et al., 2012; Blein-Nicolas and Zivy, 2016). PC is a complementary method that quantifies and compares the number of detected peptides for a given protein in the samples. Data post-processing and statistical analysis were performed by using the R package^4^ MCQR 0.4.3. Different bioinformatic pipelines were applied as indicated. For SC it involved: i) removal of proteins having <5 spectra in all samples, and ii) removal of proteins with <1.5 variation between strains. For XIC it included: i) removal of peptides with high retention time variation >20 s and peak width >200 s, ii) normalization of peptide intensities based on a reference sample, iii) removal of shared peptides, iv) removal of peptides with >5% of missing values in the whole experiment, v) peptides correlated to a reference peptide with a coefficient of correlation (*r*^2^ < 0.75) were kept for further analysis, vi) missing values of peptide intensities were imputed by replacing them with the minimum abundance obtained for this protein in the whole experiment, and vii) removal of peptides showing abundance variation <1.5 between strains. For PC: i) removal of peptides with high retention time variation >20 s and peak width >200 s, ii) removal of proteins showing <5 peaks in all samples, and iii) removal of proteins with <1.5 variation between conditions. Protein abundance changes were detected by ANOVA tests for all methods (SC, XIC, and PC), the obtained *p* values were adjusted by the Benjamini–Hochberg approach (Benjamini and Hochberg, 1995). The abundance of a protein was considered significantly variable when the adjusted p value was <0.01. Descriptive analysis of protein abundances was performed based on heatmap representations and calculated relative protein abundance ratios. Heatmaps were constructed using hierarchical clustering based on Euclidean distances. MS data are available via ProteomeXchange (Perez-Riverol et al., 2019) with the identifier PXD012672. In order to annotate the functions of proteins with detected abundance changes (adjusted *p* value <0.01), we used BioCyc database^5^ and the Gene Ontology Resource^6^ along with literature searches.

The bioinformatic tools used for proteomics analysis (X!TandemPipeline C++, MassChroQ, MCQR) are open and free resources available in the following repository: https://forgemia.inra.fr/pappso.

## Results

### Phenotypes of *S. coelicolor* A3(2) Δ*cpkO* and Δ*cpkN* Strains

Deletion of *cpkO* gene was previously shown to abolish coelimycin synthesis (along with transcription of some of *cpk* genes) on minimal and rich media supplemented with glutamate but no clear additional phenotypic effects were reported (Gottelt et al., 2010). CpkN was so far regarded as a putative activator of *cpk* cluster.

**FIGURE 1:**
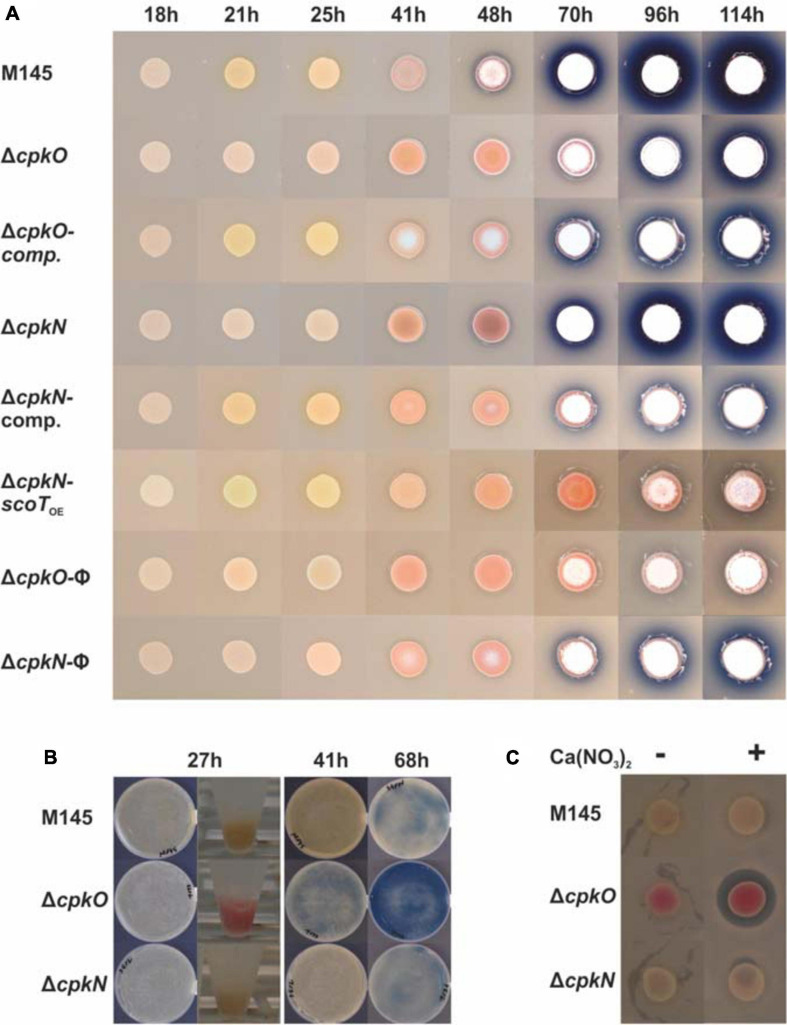
Phenotypes of *S. coelicolor* A3(2) wild-type (M145) and mutant strains cultivated on solid medium 79NG. CPK, RED, and ACT are visible as yellow, red and blue pigments, respectively. Aerial mycelium is white. **(A)** *Streptomyces* strains grown as spots; comp., complemented strains; *scoT*_OE_, overexpression of *scoT*; φ, strains with an empty plasmid. **(B)** Growth on plates covered with cellophane disks and biomass collected for proteomic analysis (in Eppendorf tubes). **(C)** Assay for calcium dependent antibiotic production. Inhibition zone around the colony of Δ*cpkO* strain in the presence of Ca(NO_3_)_2_ indicates CDA production.

Our results of phenotypic studies performed on solid rich medium without glucose (79NG) demonstrate that deletion of either *cpkO* or *cpkN* gene results in loss of coelimycin synthesis (Figures 1A,B). Complementation of both *cpkO* and *cpkN* deletions with respective genes under their native promoters (strains *cpkO*_CO_ and *cpkN*_CO_, respectively) restored CPK production. The phenotypic panel shown in Figure 1A also includes the strain Δ*cpkN*-*scoT*_OE_ – a *cpkN* deletion mutant in which the type II thioesterase ScoT gene was expressed from a strong constitutive promoter *ermEp*^∗^. Thioesterase ScoT is an editing enzyme responsible for correct functioning of the polyketide synthase and necessary for CPK production (Kotowska et al., 2014). Synthesis of CPK was restored in Δ*cpkN*-*scoT*_OE_ strain leading to the conclusion that activation of *scoT* is the sole function of CpkN in the control of *cpk* gene cluster. Transcriptional and proteomic background for this effect is discussed in more detail below.

Deletion of *cpkO* led to enhanced undecylprodigiosin and calcium dependent antibiotic synthesis in comparison to the wild-type strain M145 (Figure 1). When bacteria were grown as spots, actinorhodin production was delayed for around one day and markedly reduced in Δ*cpkO* strain (onset after 70 h of growth) in comparison to the wild-type strain (Figure 1A). Δ*cpkN* strain showed only slightly reduced ACT synthesis in these conditions. When bacteria were grown on a cellophane disk covering the surface of the agar medium (for the proteomic analysis), ACT production was clearly enhanced in Δ*cpkO* strain and not affected in Δ*cpkN* strain (Figure 1B).

### Proteomes of *S. coelicolor* A3(2) Δ*cpkO* and Δ*cpkN* Mutants Are Strongly Altered

**FIGURE 2:**
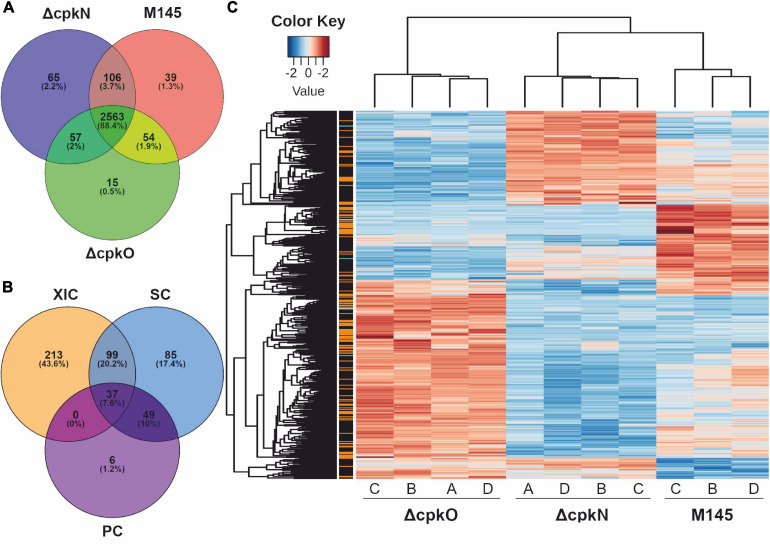
Proteomic analysis of *S. coelicolor* A3(2) wild-type (M145) and mutant strains. **(A)** Protein identifications in the analyzed strains. **(B)** Quantification of 489 proteins showing significant abundance change between the strains (ANOVA test, adjusted *p* value <0.01); methods: XIC, extracted ion chromatograms; SC, spectral count; PC, peak count. **(C)** Global heatmap representation of protein abundances with significant abundance change in function of the different strains (ANOVA test, adjusted *p* value <0.01). The method of quantification of each protein is indicated in colors on the left of the heatmap for extracted ion chromatogram (black), spectral count (orange) or peak count (cyan).

**FIGURE 3:**
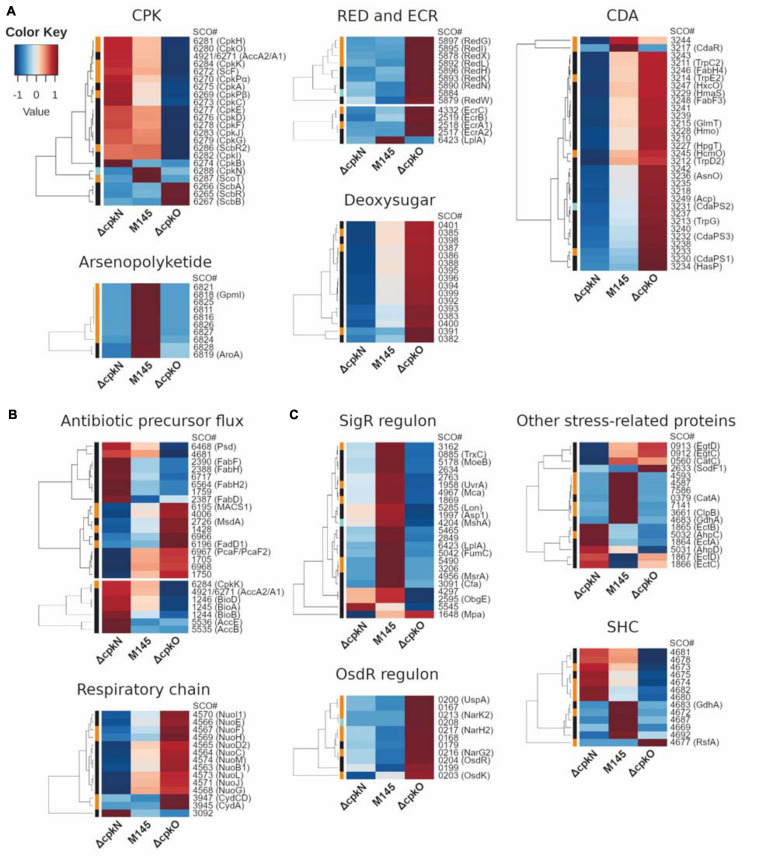
Heatmap representation of mean abundances of proteins in *S. coelicolor* A3(2) M145, *ΔcpkO* and *ΔcpkN* strain. **(A)** Proteins from specialized metabolite biosynthetic clusters; ECR, expression correlated with RED. **(B)** Proteins associated with primary metabolism. **(C)** Proteins related to stress response and others; SHC, supercoiling-hypersensitive cluster. The method of quantification of each protein is indicated in colors on the left of the heatmap for extracted ion chromatogram (black), spectral count (orange) or peak count (cyan).

Growth conditions and timing optimal for CPK production in *S. coelicolor* A3(2) occurred in cultures of medium 79NG at 27 h (Figure 1B). Based on the identified peptides (Supplementary Table S4), a total of 2,899 proteins (36% of the theoretical proteome) were identified in the studied strains M145, Δ*cpkO* (P193) and Δ*cpkN* (P196) (Supplementary Table S5). The distribution of protein identifications in the strains (Supplementary Table S6) is shown in Figure 2A. During data post-processing one sample (M145 A) was excluded from data analysis because of its dubious LC-MS/MS results, observed in principal component analysis and in peptide retention time variation (not shown). The data treatment resulted in 1862 valid proteins quantified by XIC (extracted ion chromatograms), SC (spectral counting) and/or PC (peak counting) complementary methods. When measurements by multiple methods were available, XIC result was prioritized over SC result, which in turn was prioritized over that of PC. Out of all quantified proteins, 489 showed statistically significant differences in abundance (adjusted *p* value < 0.01) between at least two of the studied strains (Supplementary Table S7). Among them, 349 proteins were quantified using XIC, 270 proteins quantified by SC and 92 quantified by complementary PC method (Figure 2B). The 489 statistically significant proteins (adjusted *p* value < 0.01) are represented in a heatmap using hierarchical clustering (Figure 2C). The replicates of each strain were grouped together by an unsupervised clustering method indicating the reproducibility of sample preparation in the experiment. The most pronounced abundance changes were observed for proteins involved in specialized metabolism, however, we also detected unexpected abundance variation in stress response and primary metabolism proteins. The effects of *cpkO* and *cpkN* deletion on specialized metabolism and specialized metabolite precursor flux are presented below, while those associated with stress response, hypoxia, amino acid metabolism, morphological differentiation and others are shown in Supplementary Table S8. The comparison of mean protein abundances for selected pathways is shown in Figure 3.

### Specialized Metabolism of *S. coelicolor* A3(2) Δ*cpkO* and Δ*cpkN* Strains

#### Coelimycin Biosynthetic Gene Cluster (*cpk*)

The abundance of nearly all detected *cpk* cluster proteins (including CpkN and ScbR2) was reduced to 0 in Δ*cpkO* as a result of the absence of the cluster activator CpkO. Only butanolide system proteins (ScbA, ScbB, and ScbR) were more abundant, presumably as a result of the absence of CpkO-dependent repressor ScbR2. Contrary to this observation, *cpk* cluster biosynthetic proteins were generally more abundant, and those of butanolide system were generally only slightly less abundant in Δ*cpkN* strain (except ScbR2 which was not changed). The exception to this general *cpk* protein increase was ScoT, which was completely absent from Δ*cpkN* strain.

Putative histidine kinase OrfB (SCO6268) and a small uncharacterized protein CpkL (SCO6285) were the only members of the *cpk* cluster which were not detected. AccA1 (SCO6271) differs by only four amino acids from AccA2 (SCO4921), therefore it is not possible to distinguish accurately between these two proteins by the shotgun proteomics method. Almost all detected peptides can be attributed to both of them. AccA1 and/or AccA2 proteins were less abundant in Δ*cpkO* and more abundant in Δ*cpkN* strain.

#### Undecylprodigiosin Biosynthetic Gene Cluster (*red*)

Perhaps the most striking difference in protein abundance profiles between the mutants and the parent strain involves the undecylprodigiosin biosynthetic gene cluster. At the time-point of 27 h, all of the detected *red* proteins were drastically more abundant in Δ*cpkO* than in the parent strain, the increase being between 4-fold for the oxidoreductase RedK to 58-fold for the type I PKS RedL (Supplementary Table S7). In Δ*cpkN* strain, the profile of *red* proteins was heterogeneous.

We have also detected proteins encoded by *ecr* (expression coordinated with *red*) genes. EcrA2/A1/B (SCO2517-SCO2519) and EcrC (SCO4332) were more abundant in Δ*cpkO* strain, which corresponds to its high *red* genes expression, while in Δ*cpkN* they were unchanged or less abundant. The only exception was EcrF, also named LplA (SCO6423), which was less abundant in both mutants. This protein, however, belongs to the SigR regulon and can also be influenced by cellular stress (Supplementary Table S8).

#### Calcium-Dependent Antibiotic Biosynthetic Gene Cluster (*cda*)

The mutants Δ*cpkN* and Δ*cpkO* presented the opposite *cda* cluster proteomic profiles in comparison to the M145 strain. Deletion of *cpkN* caused a marked decrease in the amount of all 31 detected *cda* proteins, while deletion of *cpkO* led to strong increase in the amount of 29 of them. Enhancement of CDA production in Δ*cpkO* after 27 h of growth is also reflected in an *in vivo* CDA assay (Figure 1C). Interestingly, contrary to the general cluster profiles, the putative *cda* cluster-specific activator CdaR, along with uncharacterized protein SCO3244, were less abundant in both Δ*cpkO* and Δ*cpkN* strains.

#### Arseno-Polyketide Biosynthetic Gene Cluster

Recently, evolutionary-driven natural product mining led to the identification of arseno-organic metabolite both in *S. coelicolor* A3(2) and *S. lividans* TK24. Although its exact structure has not yet been elucidated, the compound is predicted to be a polar arseno-polyketide and its synthesis was abolished by deletion of *SCO6819* gene. In our studies, all detected proteins from this cluster, including SCO6819, were virtually absent from the proteomes of Δ*cpkN* and Δ*cpkO* strains. Our results also indicate that besides the protein range SCO6812-SCO6837 (Cruz-Morales et al., 2016), SCO6811 most likely also belongs to the same cluster, as it demonstrates the same profile.

#### Other Specialized Metabolite Biosynthetic Gene Clusters

Abundances of proteins from other specialized metabolite BGCs are presented in Supplementary Table S7. Out of nine detected proteins encoded within the putative hopene and aminotrihydroxybacteriohopane (ATBH) biosynthetic gene cluster (van Keulen and Dyson, 2014), three (SCO6764, SCO6766, and SCO6768) showed significant abundance variation between the strains. While in Δ*cpkN* strain all of them were strongly increased, in Δ*cpkO* strain SCO6764 and SCO6768 were more abundant and SCO6766 was slightly less abundant in comparison to the wild-type strain M145.

Coelichelin is an atypical hydroxamate-siderophore encoded within the *cch* gene cluster, which was represented in our study by ten detected proteins, however, only four of them (SCO0492, SCO0494, SCO0495, and SCO0498) demonstrated significant abundance changes between the strains. The amount of all of them was markedly increased in Δ*cpkN* strain in comparison to M145. SCO0492 and SCO0498 were also more abundant in Δ*cpkO* strain, however, the remaining two proteins did not show abundance differences in comparison to the wild-type strain.

*SCO0381-SCO0401* gene cluster encodes proteins of an unknown deoxysugar synthesis process. All of the 16 proteins detected in this study were less abundant in Δ*cpkN* and more abundant in Δ*cpkO* in comparison to M145 strain (Figure 3A).

Interestingly, we have detected SCO1222 and SCO1223, proteins probably belonging to one transcriptional unit – having 66 and 82% identity, respectively, to the corresponding proteins of salinomycin BGC from *Streptomyces albus* (Medema et al., 2015). These proteins were both less abundant in Δ*cpkN* and much more abundant in Δ*cpkO*. They were previously prioritized for further study based on strict regulation of their respective genes’ transcription and expression profile resembling that of *act* and *red* clusters (Alam et al., 2011). As for now, the pathway that involves SCO1222 and SCO1223 has still not been resolved.

### Transcriptional Profiling of Coelimycin Biosynthetic Gene Cluster

**FIGURE 4:**
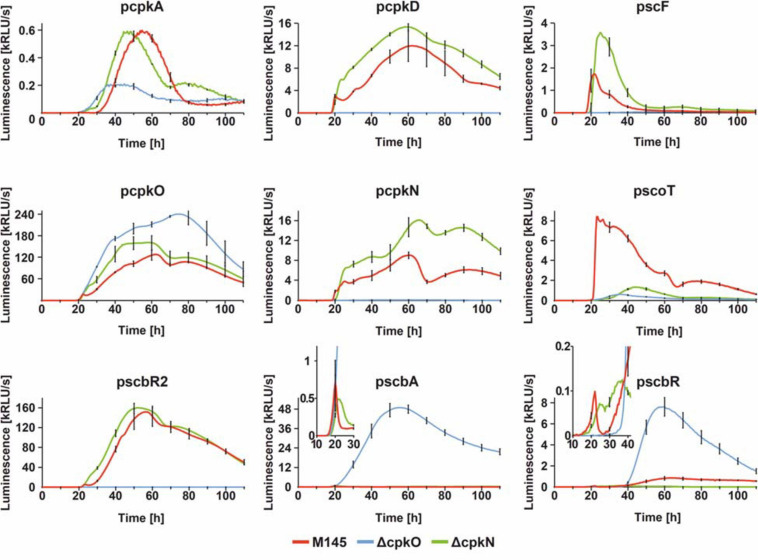
Transcriptional profiles *cpk* cluster promoters. The charts show luciferase-based reporter system measurements over 110 h of growth with sampling time of 30 min. For clarity, standard deviation was shown for every 10 h time-point. In case of pscbA and pscbR profiles, parts of the graphs were zoomed in to better visualize the differences in earlier time-points.

In order to gain an insight into the mechanism of coelimycin synthesis regulation, we employed dense time-point luciferase reporter system measurements to *cpk* cluster promoters in *S. coelicolor* A3(2) wild-type M145, Δ*cpkO*, and Δ*cpkN* strains. Bacteria were grown in an optical-bottom, white 96-well plate, on solid medium 79NG and luminescence was measured every 30 min for 110 h. Selected promoter regions included those of regulatory genes (pcpkO, pcpkN, pscbR, pscbR2, and pscbA) and structural genes (pcpkA, pcpkD, pscF, and pscoT). Fragment pcpkA is located upstream of co-transcribed genes *cpkA/cpkB/cpkC* of the modular polyketide synthase core subunits and pcpkD represents co-transcribed post polyketide tailoring genes (Chen et al., 2016). Although it was shown that *cpkO* is a part of *cpkD/cpkE/cpkF/cpkG/cpkO/cpkH* transcriptional unit, and *cpkN* is co-transcribed with its preceding gene *scoT* (Chen et al., 2016), our results show that the sequences immediately upstream of *cpkO* and *cpkN* are functional promoters. Transcription profiles are shown in Figure 4.

Observed effects of *cpkO* and *cpkN* deletion are in agreement with proteomic data. Deletion of *cpkO* gene leads to the most distinct effects. In Δ*cpkO* strain, transcription is silenced for all four analyzed biosynthetic genes (*cpkA*, *cpkD*, *scF*, and *scoT*), along with two regulatory genes *cpkN* and *scbR2*. These results indicate that CpkO is the higher-level activator of *cpk* cluster – activating other regulatory genes (*cpkN* and *ScbR2*) and possibly biosynthetic pathway genes. At the same time, transcription of the regulatory genes *scbA*, *scbR*, and *cpkO* itself is noticeably upregulated. Since it is unlikely for a SARP to act as a repressor, this effect can be attributed to the lack of ScbR2 – the repressor of *cpkO* and *scbA* (Gottelt et al., 2010; Wang et al., 2011). It is noteworthy that *cpkO* transcription level in the mutant is “resistant” to the elevated level of *cpkO* repressor *scbR*, although a slight retardation can be seen that shifts *cpkO* transcription peak at ∼60 to ∼75 h. Interestingly ∼60 h timepoint is the peaking point of *scbR* transcription. This weak repression of *cpkO* by ScbR may be a consequence of the latter protein population being complexed with GBL as a consequence of strong transcription of *scbA* in Δ*cpkO*. Binding of GBL also reverses autorepression of ScbR.

On the other hand, Δ*cpkN* strain was characterized by slightly elevated (or slightly precocious, in the case of *cpkA* and *scbR2*) transcription of most of *cpk* cluster genes, with the exception of *scbA*, *scbR*, and *scoT*. Lower *scbA* and *scbR* levels can be easily attributed to the precocious *scbR2* transcription in Δ*cpkN*. However, there is no explanation for the strong downregulation of *scoT* gene other than that CpkN is the activator of *scoT*. This statement is reinforced by phenotypic and proteomic analyses (previous sections). Weak transcription from *scoT* promoter was observed in Δ*cpkN* strain after approximately 40 h of growth, while in Δ*cpkO* strain it was completely abolished. This suggests that CpkO can activate transcription of *scoT* independently from CpkN, but this activation is too low to maintain CPK production. As for transcription upregulation of some *cpk* genes (*cpkA*, *scF*, *cpkN*, and *scbR2*) in Δ*cpkN*, we propose that it’s unlikely that CpkN has any inhibitory activity. This effect is most likely the result of the negative feedback loop driven by lack of coelimycin synthesis that results in *cpkO* upregulation, responsible for all the effects that follow (see section “Discussion”).

### ScbR and ScbR2 Interplay Within the *cpkN* Promoter

**FIGURE 5:**
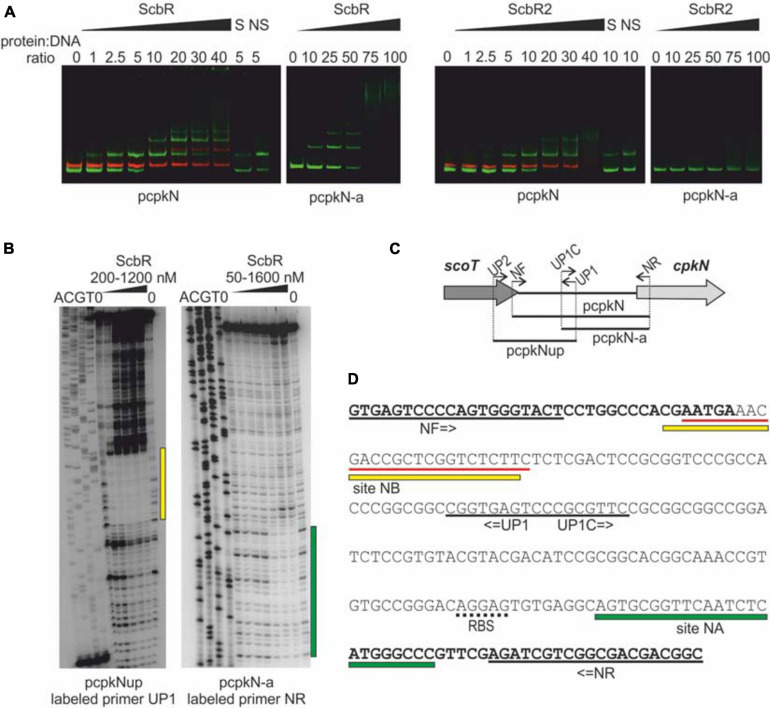
Binding of His-tagged ScbR and ScbR2 proteins to *cpkN* promoter region. **(A)** Electrophoretic mobility shift assay. Green bands – IRDye 800 labeled fragments pcpkN and pcpkN-a, red bands – IRDye 700 labeled negative control fragment MYCO, S, 10-fold excess of unlabeled specific competitor DNA (pcpkN); NS, 10-fold excess of unlabeled non-specific competitor DNA (MYCO). **(B)** Protection of *cpkN* promoter region from digestion by ScbR protein (DNase I footprint). The amplified fragments and the radiolabeled primers are indicated below the lanes. Protected regions are marked with yellow and green bars. **(C)** Schematic representation of the location of primers and fragments used for EMSA and footprint experiments. **(D)** Nucleotide sequence of pcpkN fragment. Coding sequences of *scoT* and *cpkN* genes are in bold. Predicted ribosome binding site (RBS) is underlined with a dotted line. Primer sequences are underlined with black solid lines. Regions protected by ScbR protein from **(C)** are marked with yellow and green bars. Sequence recognized by ScbR2 reported by Li et al. (2015) is underlined with red lines.

ScbR2, but not ScbR has been previously shown to bind *cpkN* gene promoter (Li et al., 2015). Here, we report identification of two ScbR binding sites within the *cpkN* promoter along with the already-reported binding site for ScbR2 (Figure 5). We observed binding of both ScbR and ScbR2 to the fluorescently labeled pcpkN promoter fragment in the EMSA assay. Shorter fragment (pcpkN-a) was bound only by ScbR (Figure 5A). Using DNase I footprinting (Figure 5B) we identified two nucleotide sequences protected from enzymatic digestion by binding of ScbR. While one of the binding sites (site NB) overlaps with that of ScbR2, the second one is exclusive to ScbR (site NA) (Figure 5D). Location of the respective DNA fragments within *cpkN* promoter was presented in Figure 5C.

### Primary Metabolism and Specialized Metabolite Precursor Flux in *S. coelicolor* A3(2) Δ*cpkO* and Δ*cpkN* Strains

In bacteria, genes of primary metabolism are usually clustered in operons, however, members of *Streptomyces* genus often possess multiple paralog genes, located at different chromosomal positions, that encode proteins with similar functions (Schniete et al., 2018). This feature hinders the analysis of primary metabolism pathways, especially in the absence of metabolomics data. Nevertheless, we were able to detect consistent patterns of primary metabolism deregulation, the most important being the changes in specialized metabolite precorsor flux (Figure 3B). Changes in other primary metabolism pathways and stress response were shown in Supplementary Table S8 and Figure 3C. A link between hypoxia and stress response is discussed below.

#### Lipid Metabolism and Polyketide Precursor Flux

Biosynthesis of both fatty acids and polyketides require the same building blocks, typically acetyl-CoA as a starter unit and acetyl-CoA derived malonyl-CoA as the most common extender unit (Hopwood and Sherman, 1990). It was shown recently that the pool of acetyl-CoA for polyketide synthesis, which takes part in the stationary phase of growth, comes from degradation of triacylglycerols accumulated earlier (Wang et al., 2020).

We observed a pattern of lipid biosynthesis proteins being generally more abundant in Δ*cpkN* and less abundant in Δ*cpkO*, while lipid degradation proteins were generally less abundant in Δ*cpkN* and more abundant in Δ*cpkO*. Biosynthesis group consisted of: SCO1759, SCO2387 (FabD), SCO2388 (FabH), SCO2390 (FabF), SCO4681, SCO6468, SCO6564 (FabH2), SCO6717 and degradation group members were: SCO1428, SCO1705, SCO1750, SCO4006, SCO6195 (MACS1), SCO6196 (FadD1), SCO6966, and SCO6968 (Kanehisa et al., 2016).

The first step of lipid degradation is the synthesis of fatty acyl-CoA. We have found two co-transcribed fatty acyl-CoA synthetases: MACS1 (SCO6195) and FadD1 (SCO6196) to be less abundant in Δ*cpkN* and more abundant in Δ*cpkO*. FadD1 is produced in the stationary phase of growth, its level was shown to be positively correlated with ACT production (Banchio and Gramajo, 2002) and it is the key enzyme for triacylglycerol degradation (Wang et al., 2020). Additional acetyl-CoA could also be supplied by MsdA (SCO2726) or SCO6967 (a PcaF homolog) (Davis and Sello, 2010) with putatively co-transcribed SCO6968 and neighboring SCO6966 (Kanehisa et al., 2016) – their abundance was generally reduced in Δ*cpkN* and increased in Δ*cpkO*.

Malonyl-CoA biosynthesis occurs through carboxylation of acetyl-CoA by an acetyl-CoA carboxylase (ACCase) (Toh et al., 1993). Acetyl-CoA carboxylase consists of two subunits (α and β chains) and relies on biotin as a cofactor. In *S. coelicolor* A3(2) there are two almost identical α subunits, AccA1 (SCO6271), encoded within *cpk* gene cluster, and essential AccA2 (SCO4921) (Rodríguez et al., 2001). We have found that ACCase α subunit(s) AccA2 and/or AccA1, β subunits AccB (SCO5535) and CpkK (SCO6284), and a small accessory protein AccE (SCO5536), were more abundant in Δ*cpkN* along with biotin biosynthesis proteins BioA (SCO1245), BioB (SCO1244), and BioD (SCO1246). Δ*cpkO* strain presented a different pattern of deregulation – the amounts of AccA2 (and/or AccA1) and CpkK were reduced and AccB and AccE remained generally unchanged, while BioA, BioB, and BioD were all less abundant. SCO1243 – another biotin synthesis protein, belonging to a different transcriptional unit than the predicted *bioBAD*, remained unchanged in both mutant strains.

Malonyl-CoA is a precursor for CPK, RED, and ACT synthesis, hence a feedback loop between their BGCs and malonyl-CoA synthesis possibly exists. Previous results indicated that in the case of *cpk* cluster it is a negative feedback loop, operating via repression of *accA2* by ScbR and ScbR2. Promoter of *accA1* was also shown to be the target for ScbR2 (Li et al., 2015). Transcription of *cpk* cluster results in high abundance of ScbR2, activated by CpkO. Here, despite drastically reduced level of ScbR2 in Δ*cpkO* strain, the level of AccA2 (and/or AccA1) is not increased, as expected, but reduced, suggesting involvement of another repressor or direct activation by CpkO. The latter hypothesis is in concordance with higher abundance of AccA2 (and/or AccA1) in Δ*cpkN* strain in which ScbR2 level is similar as in the wild type, but the level of CpkO is considerably increased.

## Discussion

Production of antibiotics and other specialized metabolites provides necessary means for bacteria to compete with other organisms in the ever-changing environmental conditions and niches. Therefore, transcription of biosynthetic genes has to be tightly controlled.

Although the subject of coelimycin synthesis regulation has received much attention in the past, it was mainly focused on the roles of TetR-like regulators ScbR and ScbR2, presumably because of their ability to affect production of many antibiotics and thus provide a link between multiple specialized metabolism pathways. On the contrary, regulation of *cpk* cluster expression by SARP proteins CpkO and CpkN has been partially neglected because of their predicted mode of action as direct activators of biosynthetic genes, operating within the boundaries of their own cluster.

Previous studies have shown that CpkO is the activator necessary for transcription of *cpk* cluster (Takano et al., 2005) and that deletion of its gene abolishes coelimycin synthesis (Gottelt et al., 2010). However, no DNA binding sites or a comprehensive list of target genes were determined for this regulator, making it impossible to tell whether its action is direct or indirect. The second SARP regulator, CpkN, was so far regarded as a putative activator of *cpk* cluster, however, with no experimental evidence to this hypothesis.

Our findings revealed that both CpkO and CpkN are activators required for CPK biosynthesis, but their regulatory effects extend to other pathways of specialized metabolism and beyond.

### CpkO and CpkN Activate Coelimycin Biosynthetic Cluster in a Cascade Manner and Are Both Directly Controlled by the Butanolide System Repressors

In this work we have shown that, just like CpkO, CpkN is also required for CPK production (Figure 1). CpkO activates most of *cpk* cluster genes i.e. *scbR2* as well as structural genes, however it is not an autoactivator (Figures 3, 4). Because the levels of ScoT and CpkN were drastically decreased in Δ*cpkO* strain and ScoT was also absent from Δ*cpkN*, despite the presence of CpkO in this strain, we propose that CpkO is the activator of *cpkN* and CpkN is the activator of *scoT*. Indeed, complementation of *cpkN* deletion with *scoT* gene restored CPK production. ScoT is a type II thioesterase that was previously shown by our group to be necessary for coelimycin synthesis. It was proposed to maintain PKS activity by removal of non-reactive acyl residues blocking the “assembly line” (Kotowska et al., 2014).

CpkO is directly repressed by GBL receptor ScbR and pseudo GBL receptor ScbR2. There are two binding sites in *cpkO* promoter region, site OA recognized only by ScbR and site OB recognized by both ScbR and ScbR2 (Takano et al., 2005; Wang et al., 2011). Our results indicate that *cpkN* promoter region is bound by the two regulators in the same manner. We identified two binding sites of ScbR protein, site NA and site NB (Figure 5D). Site NB corresponds to the binding site of ScbR2 protein reported by Li et al. (2015). However, the consensus motifs of ScbR and ScbR2 target sequences proposed in that work cannot be readily found within NA and NB sites. The existence of overlapping ScbR and ScbR2 binding sites in *cpkO* and *cpkN* promoters suggests regulatory checkpoint in CPK synthesis by competition of the two proteins, or perhaps the action of ScbR and ScbR2 at different time.

Sun et al. (2017) postulated a positive feedback loop in which coelimycin or its precursor could bind to ScbR2 homolog from *S. lividans* TK24, leading to derepression of *cpk* promoters. The experiment was based on indigoidine reporter gene under control of *scoT*-homolog promoter, which was assumed to be a target for ScbR2-homolog despite no experimental evidence. Overexpression of *cpkO*- and, particularly, *cpkN*-homolog genes led to production of indigoidine, explained as a consequence of CPK binding to ScbR2-homolog and derepression of *scoT*-homolog. Our data provide alternative explanation for the observed effect. The reason of indigoidine production could be activation of *scoT*-homolog promoter by CpkN- and CpkO-homologs rather than its derepression by CPK molecule binding to ScbR2-homolog. Indeed, according to our results, CpkN is a stronger activator of *scoT* than CpkO, which corresponds to indigoidine levels observed by Sun et al. The potential signaling role of CPK, including determination of which CPK intermediate is the active molecule, requires further studies.

**FIGURE 6:**
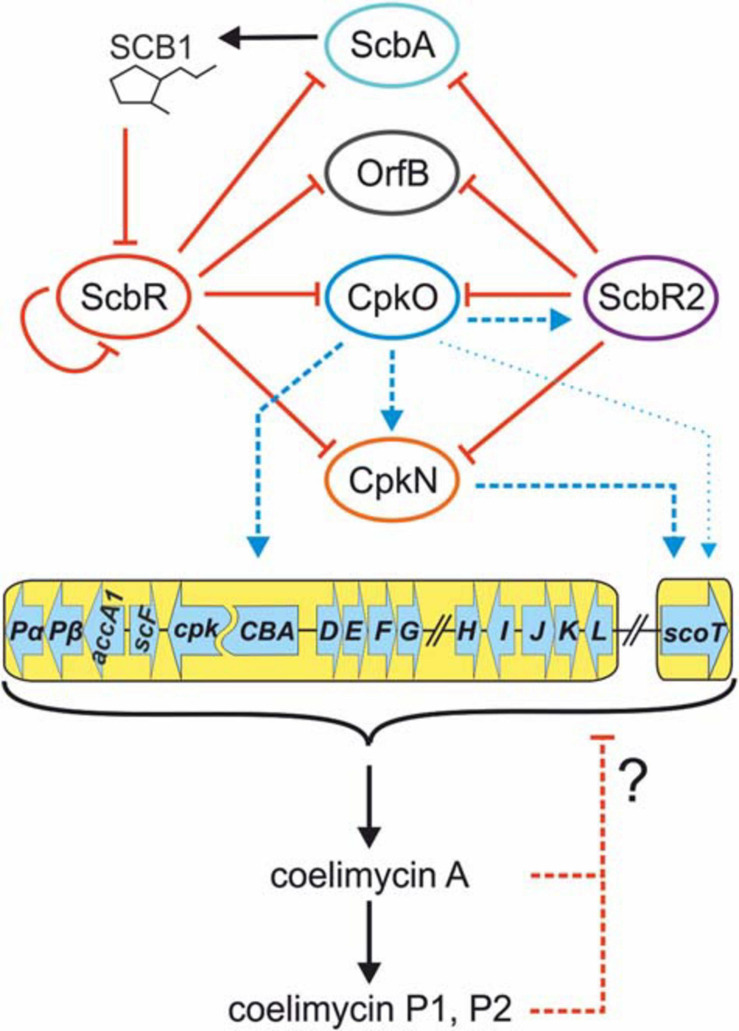
Updated mechanism of coelimycin BGC regulation by cluster-situated regulators. Black arrows indicate γ-butyrolactone SCB1 and CPK production. Solid red lines ending with bars indicate inhibition of ScbR by SCB1 and direct repression by promoter binding. Dashed red lines indicate putative negative feedback loop exerted by coelimycin. Blue lines ending with arrows denote transcription activation. Thin blue arrow indicates weak activation of *scoT* transcription by CpkO independent from CpkN. Dashed lines imply an indirect or unknown regulatory mechanism (unknown binding site of the activator). CPK biosynthetic genes are marked with a yellow background. Regulatory genes are omitted from the cluster scheme for clarity.

The updated mechanism of coelimycin synthesis regulation is shown in Figure 6.

### Decreased Oxidative Stress Response Is Correlated With Increased Hypoxia Response in CpkO- and CpkN-Deficient *S. coelicolor* A3(2)

Proteins indicative of hypoxia-like state (the OsdR dormancy regulon proteins together with its activator OsdR itself) were more abundant in both deletion mutants, being the most abundant in Δ*cpkO*. On the other hand, abundance of direct target proteins of SigR, the sigma factor governing the major oxidative stress response in *S. coelicolor* A3(2), along with many other stress-related proteins, was decreased in both deletion mutants, being the least abundant in Δ*cpkO* strain (Figure 3, Supplementary Table S8). It seems plausible that these effects are linked together – production of reactive oxygen species increases with oxygen concentration (Imlay, 2019), hence Δ*cpkO* and Δ*cpkN* mutants in hypoxia would generally receive less damage from oxidative stress.

A transcriptomic study showed that, in addition to the dormancy/hypoxia related genes, OsdR activates a number of developmental and general stress response genes, including many of SigR targets, as well as genes related to sulfur metabolism and cysteine biosynthesis (Urem et al., 2016). Here, despite increased levels of OsdR, abundance of cysteine metabolism proteins was reduced in Δ*cpkO* and Δ*cpkN* mutants, similarly to SigR regulon proteins (see Supplementary Table S8), suggesting involvement of another control mechanisms.

*Streptomyces* life cycle involves formation of a dense vegetative mycelium which may locally experience hypoxia (Sawers et al., 2019). An early, compartmentalized mycelium (MI) is transformed to a multinucleated substrate mycelium (MII without the hydrophobic layer) which starts specialized metabolite synthesis and further develops into aerial mycelium (MII with a hydrophobic layer) and into spore chains (Yagüe et al., 2013). The MI/MII transition involves a programmed cell death process accompanied by the appearance of, among others, oxidative stress response proteins (Manteca et al., 2006). The authors consider the oxidative stress either as the cause or the consequence of programmed cell death. Unexpectedly, four *cpk* cluster proteins (CpkPα, CpkD, CpkE, and CpkI) were identified in the dead (but not live) cells of the early mycelium MI (Manteca et al., 2006). Gene expression in the two types of mycelia differs greatly (Yagüe et al., 2013). Transcriptomic studies revealed that expression of the majority of *cpk* genes was higher in MI than in MII (Yagüe et al., 2013), while expresion of the repressor ScbR2 was upregulated in MII.

There is a possibility of oxidative stress response induction by the reactive, bis-epoxide compound coelimycin A. Perhaps coelimycin production timing (around vegetative-aerial mycelium transition phase; Gottelt et al., 2010; Nieselt et al., 2010) is non-contingent and CPK A is an effector molecule synthesized upon a “high density mycelium” signal (sensed by γ-butyrolactone receptor ScbR), that oxidatively damages early vegetative mycelium MI for the purpose of aerial hyphae formation. Interestingly, RED production was recently shown to be associated with the death round of vegetative mycelium. However, the evidence suggests that the synthesis is the consequence rather than the trigger for programmed cell death (Tenconi et al., 2018). Protein abundance changes indicating increased hypoxia and reduced oxidative stress were more pronounced in Δ*cpkO* than in Δ*cpkN* strain. This may be explained by a possible residual production of CPK A in Δ*cpkN* cells, the level of which is too low to turn into a visible amount of the yellow pigment. However, direct detection of CPK A is highly challenging as it is predicted to be unstable and has not been detected experimentally so far.

### CpkO and CpkN Act as Pleiotropic Regulators of Specialized Metabolism

Deletion of *cpkO* and *cpkN* genes influenced both well-studied and cryptic specialized metabolite BGCs. The strains often produced opposite patterns of deregulation for a given BGC, suggesting that other clusters respond to regulation by CpkO and/or CpkN rather than just the presence or absence of CPK, synthesis of which utilizes polyketide precursors.

In a previous report (Gottelt et al., 2010) expression of *actII-ORF4* and *redD* genes was found to be precocious in *cpkO* deletion mutant in comparison to the wild-type strain M145 grown in liquid SMM medium. This suggests the possibility of an increased actinorhodin and undecylprodigiosin production by the mutant, however, the authors did not mention any differences in these pigments production on any of the tested media.

In this study RED production was indeed increased in Δ*cpkO* strain (Figure 1) and this phenotype corresponds well to the proteomic background (Figure 3). The heterogeneous profiles of *red* proteins in Δ*cpkN* resulted in no visible change in RED production by this strain. As anticipated, proteomic profiles of *ecr* proteins matched those of *red* proteins in a given strain, further emphasizing their close co-regulation.

Surprisingly, in contrast to other pigments, actinorhodin production was affected by growth on a cellophane disk used to enable biomass collection from the agar plate (Figures 1A,B). Δ*cpkO* grown directly on the 79NG medium produced considerably less ACT than the other strains, while on cellophane ACT synthesis was strongly upregulated in this strain. This observation underpins the importance of careful consideration of growth conditions when analyzing *Streptomyces* metabolism. ACT biosynthesis is activated relatively late in the culture development, therefore proteins from its biosynthetic cluster were not detected in the samples from the 27 h of growth.

We have seen completely opposite calcium-dependent antibiotic BGC protein abundance profiles in both mutants (Figure 3). The cluster was upregulated in Δ*cpkO* and downregulated in Δ*cpkN*, with the exception of the SARP activatory protein CdaR, which was less abundant in both mutants. Similar CdaR levels suggest CdaR-independent *cda* cluster regulation by an unknown mechanism, perhaps involving two-component system AbsA1/A2, encoded within the *cda* cluster (McKenzie and Nodwell, 2007). Enhanced CDA production by Δ*cpkO* strain was confirmed by a plate test (Figure 1C).

We observed a well-represented and consistent pattern of almost complete arsenopolyketide BGC silencing in Δ*cpkO* and Δ*cpkN* (Figure 3). The cluster contains five putative ArsR-family regulators, none of which were detected in our study. Since both mutants lack CpkN protein and the ability to produce coelimycin, we propose that either CpkN might be the activator (direct or indirect) of the arseno-organic metabolite BGC or coelimycin itself may facilitate its activation.

### Deletion of the Major Coelimycin Synthesis Activator Directs the Precursor Flux to the Production of Other Antibiotics

Deletion of *cpkO* gene caused a profound induction of actinorhodin, undecylprodigiosin and calcium-dependent antibiotic production when grown on 79NG medium overlaid with a cellophane disk (Figure 1). A very recent discovery linking triacylglycerol (TAG) usage with polyketide synthesis levels (Wang et al., 2020) provides an excellent background explanation for ACT overproduction in Δ*cpkO* strain. The proteomic profile of generally decreased fatty acid biosynthetic protein abundance and increased fatty acid degradation protein abundance, including that of FadD1 (SCO6196) at 27 h time-point (Figure 3), implies early activation of accumulated cellular TAG pool degradation in Δ*cpkO*. Fatty acyl-CoA synthetase SCO6196 is the key enzyme responsible for TAG mobilization and its levels correlate well with polyketide synthesis levels. TAG degradation provides acetyl-CoA for polyketide production along with reducing power and ATP that inhibit enzymes of tricarboxylic acid cycle, directing even more acetyl-CoA to ACT synthesis (Wang et al., 2020). Δ*cpkN* strain showed the opposite lipid biosynthesis/degradation profile (Figure 3) and hence it did not exceed antibiotic production capabilities of the wild-type strain (Figure 1B).

It is uncertain why deletion of *cpkO* results in increased calcium-dependent antibiotic and undecylprodigiosin production. It is worth noting that tryptophan is a precursor for CDA synthesis and this process has to depend on primary metabolism TrpAB synthase (SCO2036 and SCO2037) as *cda* cluster itself does not encode any tryptophan synthases (Hojati et al., 2002). Indeed, TrpAB synthase was overproduced in Δ*cpkO*. Undoubtedly, RED synthesis requires malonyl-CoA, derived from acetyl-CoA, plenty of which should be available in the intracellular environment of the mutant. Perhaps a complementary underlying reason for all of the described antibiotic production phenotypes in Δ*cpkO* is the overproduction of *S*-adenosylmethionine by MetK (see Supplementary Table S8 – cysteine and methionine metabolism). *S*-adenosylmethionine is a known ACT, RED, and CDA overproduction inducer (Okamoto et al., 2003). Even the slightest alterations in growth conditions seem to affect antibiotic production. When grown directly on 79NG medium, our strains showed the same CPK and RED production phenotypes as in the case of growth on cellophane-overlaid 79NG but ACT synthesis levels differed between conditions (Figure 1). This is probably caused by different rate of signaling molecule diffusion into the medium.

What is clear is that once activated, respective specialized metabolite production is not simply overtaking the productive repertoire of the cell by means of competition for available precursors. Biosynthetic gene clusters not only respond to the outside environmental stimuli but also shape the intracellular conditions and thus communicate with each other. Means to this communication are not clear. This cross-talk may be realized by cluster-situated regulators modulating primary metabolism or directly acting on other BGCs or pleiotropic regulators. Even the products of the clusters themselves may have regulatory functions. Coupling metabolic flux analysis with regulatory network models presents an exciting challenge and its outcome will profoundly facilitate industrial antibiotic-producing strain engineering.

## Data Availability Statement

The proteomics data presented are available via The Proteome Xchange Consortium with the Project Accession PXD012672. The bioinformatic tools used for proteomics analysis (X!TandemPipeline C++, MassChroQ, MCQR) are open and free resources available in the following repository: https://forgemia.inra.fr/pappso.

## Author Contributions

KP, BB, and MK designed the research and analyzed the results. KP and BB acquired funds. BB, AM-O, MK, MŚ, and JQ performed the experiments. AM-O and BB performed statistical analysis of the proteomic data, supervised by CH. BB drafted the manuscript. BB, AM-O, MK, CH, and KP wrote and revised the manuscript. All authors contributed to the article and approved the submitted version.

## Conflict of Interest

The authors declare that the research was conducted in the absence of any commercial or financial relationships that could be construed as a potential conflict of interest.

## Footnotes

1 http://www.strepdb.streptomyces.org.uk

2 http://www.thegpm.org/TANDEM/

3 http://www.uniprot.org

4 http://pappso.inra.fr/en/bioinfo/mcqr/

5 https://biocyc.org

6 https://geneontology.org
