# Supplementary material for: Coelimycin Synthesis Activatory Proteins Are Key Regulators of Specialized Metabolism and Precursor Flux in Streptomyces coelicolor A3(2)
Source: Front Microbiol. 2021 Apr 9;12:616050. doi: 10.3389/fmicb.2021.616050 (PMC8062868; doi:10.3389/fmicb.2021.616050)
Supplement: Supplementary file 1 [file Table_1.docx]

Table S1. Bacterial strains used in this work

| **Strain** | **Relevant genotype or description** | **Source or reference** |
| --- | --- | --- |
| ***Escherichia coli*** | | |
| DH5α | F^-^ endA1 glnV44 thi-1 recA1 relA1 gyrA96 deoR nupG Φ80dlacZΔM15 Δ(lacZYA-argF)U169, hsdR17(r_K_^-^ m_K_^+^), λ^–^ | Promega |
| BL21(DE3)pLysS | F-, *ompT*, *hsdSB* (rB-, mB-), *gal*, *dcm* (DE3), pLysS (Cam^R^) | Promega |
| BW25113/pIJ790 | *lacI*^+^*rrnB*_T14_ Δ*lacZ*_WJ16_ *hsdR*514 Δ*araBAD*_AH33_ Δ*rhaBAD*_LD78_ *rph-1* Δ*(araB–D)567* Δ*(rhaD–B)568* Δ*lacZ4787*(::*rrnB-3*) *hsdR514* *rph-1* pIJ790  Recombineering strain harbouring arabinose-inducible RED genes on the plasmid pIJ790. | (Gust et al., 2003) |
| ET12567/pUZ8002 | strain for conjugal transfer of DNA from *E. coli* to *Streptomyces* (*dam* *dcm* *hsdS* Cam^R^ Tet^R^ on the bacterial chromosome; *tra* Kan^R^ RP4 23 on pUZ8002) | (Kieser et al., 2000) |
| ***Streptomyces coelicolor* A3(2)** | | |
| M145 | wild type strain, *S. coelicolor* A3(2) (SCP1^-^ SCP2^-^) | (Bentley et al., 2002) |
| *∆cpkO* (P193) | SCP1^-^ SCP2^-^ *cpkO::aac3(IV)* | This work |
| *∆cpkO-φ* (P194) | SCP1^-^ SCP2^-^ *cpkO::aac3(IV)* pIJ10257 | This work |
| *cpkO_CO_* (P195) | SCP1^-^ SCP2^-^ *cpkO::aac3(IV)* pIJ10257-cpkO_CO_ | This work |
| *∆cpkN* (P196) | SCP1^-^ SCP2^-^ *cpkN*::Tn5062 | This work |
| *∆cpkN-φ* (P197) | SCP1^-^ SCP2^-^ *cpkN*::Tn5062 pIJ10257 | This work |
| *cpkN_CO_* (P198) | SCP1^-^ SCP2^-^ *cpkN*::Tn5062 pIJ10257-cpkN_CO_ | This work |
| *∆cpkN-scoT_OE_* (P199) | SCP1^-^ SCP2^-^ *cpkN*::Tn5062 pIJ10257-scoT_OE_ | This work |
| M145, P193 and P196-derivatives for luciferase reporter assay | The strains listed above harbouring pFLUXH derivatives containing sequences of different *cpk* promoters: pcpkA, pcpkD, pcpkO, pscoT, pcpkN, pscF, pscbA, pscbR and pscbR2 | This work |

Table S2. Plasmids/constructs used in this work. PCR fragments were routinely cloned into either pGEM-T Easy or pTZ57R/T, sequenced, and recloned into appropriate restriction sites of final vectors.

| **Name** | **Relevant genotype or description** | **Source or reference** |
| --- | --- | --- |
| pGEM-T Easy | T-vector for direct cloning of PCR products (Amp^R^) | Promega |
| pTZ57R/T | T-vector from InstT/A Cloning kit for direct cloning of PCR products (Amp^R^) | Fermentas (Thermo Scientific) |
| pTZ-MYCO | pTZ57R/T containing *Mycobacterium smegmatis* (NC_008596.1) intergenic region between MSMEG_6643 and MSMEG_6645 amplified with primer pair MF (CGTAGGCGAGGGCCTATGAA) and MR (CGCGATCTTCCACGCGAGAT) | gift from J. Hołówka (unpublished) |
| pET28a(+) | Novagen pET system overexpression plasmid | Novagen |
| pET28a(+)-scbR2 | pET28a(+) containing *scbR2* gene sequence amplified with primer pair R2_HE_F, R2_HE_Rv and cloned into NdeI, XhoI sites. | This work |
| pD01-HN-scbR | pET28a(+) containing *scbR* gene sequence amplified with primer pair ScbREXFW, ScbREXRV and cloned into NdeI, EcoRI sites | This work |
| St1G7 | SuperCos1 cosmid carrying fragment of  *S. coelicolor* A3(2) chromosome encompassing part of *cpk* gene cluster (bp 6905834 to 6947687) | http://www.strepdb.streptomyces.org.uk |
| St1G7-cpkO_DM_ | St1G7 cosmid, in which *cpkO* gene sequence was replaced (by means of PCR-targeting) with an apramycin resistance gene *aac(3)IV* amplified using primers CpkODM-Fw, CpkODM-Rv. Recombineering was performed by PCR-targeting in *E.coli* BW25113/pIJ790 | This work |
| 11B05.G04 | Transposon-mutagenised cosmid in which *cpkN* sequence was disrupted with Tn5062 transposon containing *aac(3)IV* casette. | (Fernandez-Martinez et al., 2011) |
| pIJ10257 | ΦBT1 integrating overexpression plasmid containing strong constitutive promter *ermEp*.* | (Hong et al., 2005) |
| pIJ10257XermEp | pIJ10257 derivative lacking *ermEp** promoter. pIJ10257 was cut with KpnI, HindIII, its ends were blunted with polymerase T4 and autoligated. | This work |
| pIJ10257-cpkO_CO_ | pIJ10257XermEp containing sequence of *cpkO* gene with its native promoter (amplified with primers CpkO_c257_RED_Eco105I_F, CpkO_c257_RED_Eco105I_R) digested with Eco105I and cloned into PvuII site of the plasmid. | This work |
| pIJ10257-cpkN_CO_ | pIJ10257 containing sequence of *cpkN* gene with its native promoter (amplified with primers CPKN_KHp, CPKN_KHR) cloned into KpnI, XhoI sites. | This work |
| pIJ10257-scoT_OE_ | pIJ10257 containing sequence of *scoT* gene (amplified with primers TE-K-Hind, TE-P-Nde) cloned under the strong constitutive promoter *ermEp** - sites NdeI, HindIII | This work |
| pFLUXH | ΦBT1 integrating reporter plasmid with a promoterless luciferase operon *luxCDAEB* and hygromycin resistance cassette | (Szafran et al., 2016) |
| pFLUXH-pcpkA  pFLUXH-pcpkD | pFLUXH-derivatives containing promoter sequence  pcpkA/pcpkD (amplified with primers p6275_Nde, p6276_Nde) cloned into NdeI site and selected for desired insert direction using luxout primer. | This work |
| pFLUXH-pcpkO | pFLUXH-derivative containing *cpkO* promoter sequence  pcpkO (amplified with primers p6280_Nde, KSO-FW) cloned into NdeI, BamHI* sites. | This work |
| pFLUXH-pcpkN | pFLUXH-derivative containing *cpkN* promoter sequence  pcpkN (amplified with primers p6288_Nde, CPKN_KHp) cloned into NdeI, BamHI* sites. | This work |
| pFLUXH-pscF | pFLUXH-derivative containing *scF* promoter sequence pscF (amplified with primers accA1-Rv, p6272_Nde) cloned into NdeI, BamHI* sites. | This work |
| pFLUXH-pscoT  pFLUXH-pscbR2 | pFLUXH-derivatives containing promoter sequence  pscoT/pscbR2 (amplified with primers p6287_Nde, p6286_Nde) cloned into NdeI site and selected for desired insert direction using luxout primer. | This work |
| pFLUXH-pscbA | pFLUXH-derivative containing *scbA* promoter sequence  pscbA (amplified with primers scbA_lux, p6266_Nde) cloned into NdeI, BamHI* sites. | This work |
| pFLUXH-pscbR | pFLUXH-derivative containing *scbR* promoter sequence  pscbR (amplified with primers SCBA-FW, p6265_Nde) cloned into NdeI, BamHI* sites. | This work |
| pET28-scbR2 | pET28a(+)-derivative containing *scbR2* gene sequence amplified with primers R2_HE_F, R2_HE_Rv, digested with PagI, XhoI and cloned into NdeI, XhoI sites of the plasmid. | This work |
| pD01-HN-scbR | pET28a(+)-derivative containing *scbR* gene sequence amplified with primers ScbREXFW, ScbREXRV, digested with NdeI, EcoRI and cloned into NdeI, EcoRI sites of the plasmid. | This work |

*BamHI site flanking the insert in pTZ57R/T was used to cut out the insert for further cloning into pFLUXH

Table S3. Primers used in this work. Restriction sites are in bold.

| **Name** | **Sequence 5’-3’** | **Restriction sites** | **Application/amplified fragment** |
| --- | --- | --- | --- |
| CPKN_KHp | **AGATCTGGTACC**GTGGCGCGAGCACACCAC | BglII, KpnI | pKH3prom |
| CPKN_KHR | **CTCGAGAAGCTTCTAGA**CCGGCCGGGTCGAGATCG | XhoI, HindIII, XbaI |  |
| CpkO_c257_RED_Eco105I_F | GATAATTTATCACCGCAGATGGTTACCTCGCCTCTGACC**TACGTA**CCGTCCCGGCGGTCGCCGGA | Eco105I | pIJ10257XermEp-cpkO_CO_ |
| CpkO_c257_RED_Eco105I_R | ACTCTAGTTAATTAATCACTCGAGATCTCATATGGGGCC**TACGTA**TCAGATCGCCCCGCCTCCG | Eco105I |  |
| TE-K-Hind | TTTTTTT**AAGCTT**GTCGTACGTACACGGA | HindIII | pIJ10257-scoT_OE_ |
| TE-P-Nde | TTTTTTTTT**CATATG**GGAAGTGACTGGTT | NdeI |  |
| CpkODM-Fw | TTTCGGATGCTCGGTCCACTCGAGGTGTTGTCCGGCGAGATTCCGGGGATCCGTCGACC | - | St1G7-cpkO_DM_ |
| CpkODM-Rv | GACGGCGGACCGCGGGCGGGCTCGGAGCAGCGGGGGTCATGTAGGCTGGAGCTGCTTC | - |  |
| p6275_Nde | **CATATG**CGGCTGCCCTTTCCTGGCTGT | NdeI | pFLUXH-pcpkA  pFLUXH-pcpkD |
| p6276_Nde | **CATATG**GATTTACTCTCCTTCGACAAG | NdeI |  |
| p6280_Nde | **CATATG**TCCCCCAGTCCTGCACGCTGT | NdeI | pFLUXH-pcpkO |
| KSO-FW | ATCATCCGGGACACCGACGGA | - |  |
| p6288_Nde | **CATATG**CTCACACTCCTGTCCCGGCAC | NdeI | pFLUXH-pcpkN |
| CPKN_KHp | **AGATCTGGTACC**GTGGCGCGAGCACACCAC | BglII, KpnI |  |
| accA1-Rv | GGCGATGAGCACCTTGCGCA | - | pFLUXH-pscF |
| p6272_Nde | **CATATG**CGAACCTCCGTGAGAACAAGA | NdeI |  |
| p6287_Nde | **CATATG**CTTTTCCCCTTACCGTTCGAC | NdeI | pFLUXH-pscoT  pFLUXH-pscbR2 |
| p6286_Nde | **CATATG**GTGCTCCGTGGTCGCGATCGT | NdeI |  |
| scbA_Lux | CAAGCGGTGACAGAACAACA | - | pFLUXH-pscbA |
| p6266_Nde | **CATATG**TCCCCCCCAGGAATCATGTGA | NdeI |  |
| SCBA-FW | TATCCAGCTGACCGGGAACGC | - | pFLUXH-pscbR |
| p6265_Nde | **CATATG**TGCCTCCTTGTTCATGTCTCC | NdeI |  |
| luxout | GCTCTCGGGGAAGATCTCGAC | - | verification of insert orientation in pFLUXH |
| R2_HE_F | AAAAAA**TCATGA**CCAAGCAGGAG | PagI | pET28-scbR2 |
| R2_HE_Rv | AAAAAA**CTCGAG**GTGCGGCGC | XhoI |  |
| ScbREXFW | **GGATCCCATATG**GCCAAGCAGGACC | BamHI, NdeI | pD01-HN-scbR |
| ScbREXRV | **GAATTC**TCA**AAGCTT**GTCCTTCCCGGTCGGTGC | EcoRI, HindIII |  |
| NF | GTGAGTCCCCAGTGGGTACT | - | pcpkN, pcpkN-a and pcpkN-up fragments as shown in Fig. 5. |
| NR | GCCGTCGTCGCCGACGATCT | - |  |
| UP1 | GAACGCGGGACTCACCG | - |  |
| UP1C | CGGTGAGTCCCGCGTTC | - |  |
| UP2 | **GGATCC**GACCTGAGGGTGTT | BamHI |  |
| pTZBAM700  (pTZBAM800) | IRDye-ATGCAGGCCTCTGCA | - | amplification and IRDye* labeling of fragments for EMSA cloned in pTZ57R/T |
| pTZXBA700  (pTZXBA800) | IRDye-TCGGTACCTCGCGAA | - |  |

* IRDye 700 (ex. 685 nm, em.: 700 nm); IRDye 800 (ex. 785 nm, em. 800 nm)
